# Cardiovocal Syndrome Secondary to Thoracic Aortic Aneurysm: An Old Sign Revisited

**DOI:** 10.7759/cureus.10087

**Published:** 2020-08-27

**Authors:** Tushar Agarwal, Jyothi Vijay, Basant Kumar, Sourabh Agstam

**Affiliations:** 1 Cardiology, Vardhman Mahavir Medical College and Safdarjung Hospital, New Delhi, IND; 2 Cardiology, Internal Medicine, Postgraduate Institute of Medical Education & Research, Chandigarh, IND; 3 Cardiology, Postgraduate Institute of Medical Education & Research, Chandigarh, IND

**Keywords:** ortner’s syndrome, thoracic aortic aneurysm, left recurrent laryngeal nerve, hoarseness of voice, dysphagia

## Abstract

A 56-year-old male, chronic smoker, presented with persistent dry cough, hoarseness of voice and difficulty in swallowing. Indirect laryngoscopy revealed left vocal cord paralysis. Further evaluation revealed eccentric saccular aneurysms arising from the aortic arch and descending thoracic aorta, compressing the trachea, esophagus, left atrium and left recurrent laryngeal nerve. The patient was diagnosed with Ortner's syndrome which is an uncommon presentation of aortic aneurysm. He awaits an endovascular aorta aneurysm repair.

## Introduction

The vocal cords are necessary for phonation. The muscles of the larynx are mainly supplied by the recurrent laryngeal nerves (RLN), which are the branches of the vagus nerve. The anatomical course of right and left recurrent laryngeal nerve is different. The left RLN loops around the aortic arch and the right RLN loops around the right subclavian artery, and then ascends in the tracheo-esophageal groove. Paralysis of vocal cords can present with symptoms like hoarseness of voice and loss of pitch [1].

Norbert Ortner, an Austrian physician, was the first to describe hoarseness of voice in a patient with mitral stenosis and left atrial enlargement in 1897 [2]. Since then, the term Ortner’s syndrome has been used to describe cardiovocal syndrome caused by left recurrent laryngeal nerve (LRLN) paralysis from various cardiac, aortic, or pulmonary pathology [1, 2]. We present an index case with symptoms suggestive to malignant etiology, and on evaluation, found to have cardiovocal syndrome by aortic saccular aneurysm. The patient was advised for endovascular aortic repair of aortic aneurysm.

## Case presentation

**Figure 1:**
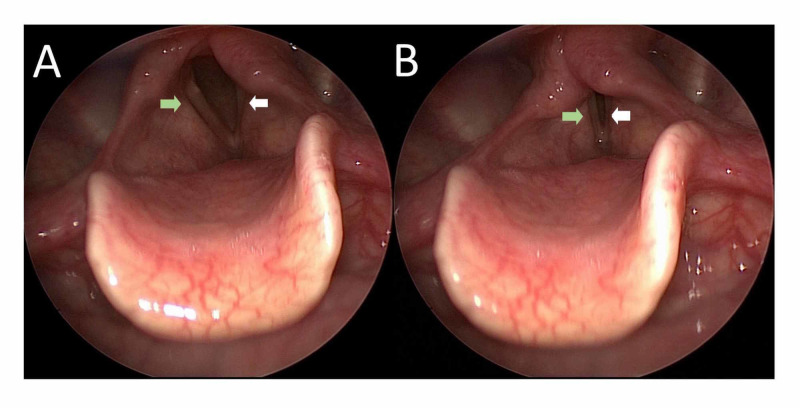
Indirect video laryngoscopy still image showing paralyzed left vocal cord (white arrow) in paramedian position and compensatory movement of right vocal cord (green arrow) during abduction (A) and adduction (B).

A 56-year-old male, labourer by occupation, was referred to the cardiology outpatient department from the otorhinolaryngology department for evaluation of a bulge adjacent to the aortic area on chest X-ray. He had a history of dry cough, thickness of voice and dysphagia for one year, associated with significant weight loss. He had a history of smoking in form of bidi 20 pack year. Indirect laryngoscopy revealed left vocal fold paralysis at the paramedian position (Figure 1, Video 1).

**Video 1:** Indirect video laryngoscopy showing fixed left vocal cord (white arrow) in paramedian position with compensatory movement of right vocal cord (green arrow) during adduction and abduction, suggestive of left vocal cord paralysis.

**Figure 2:**
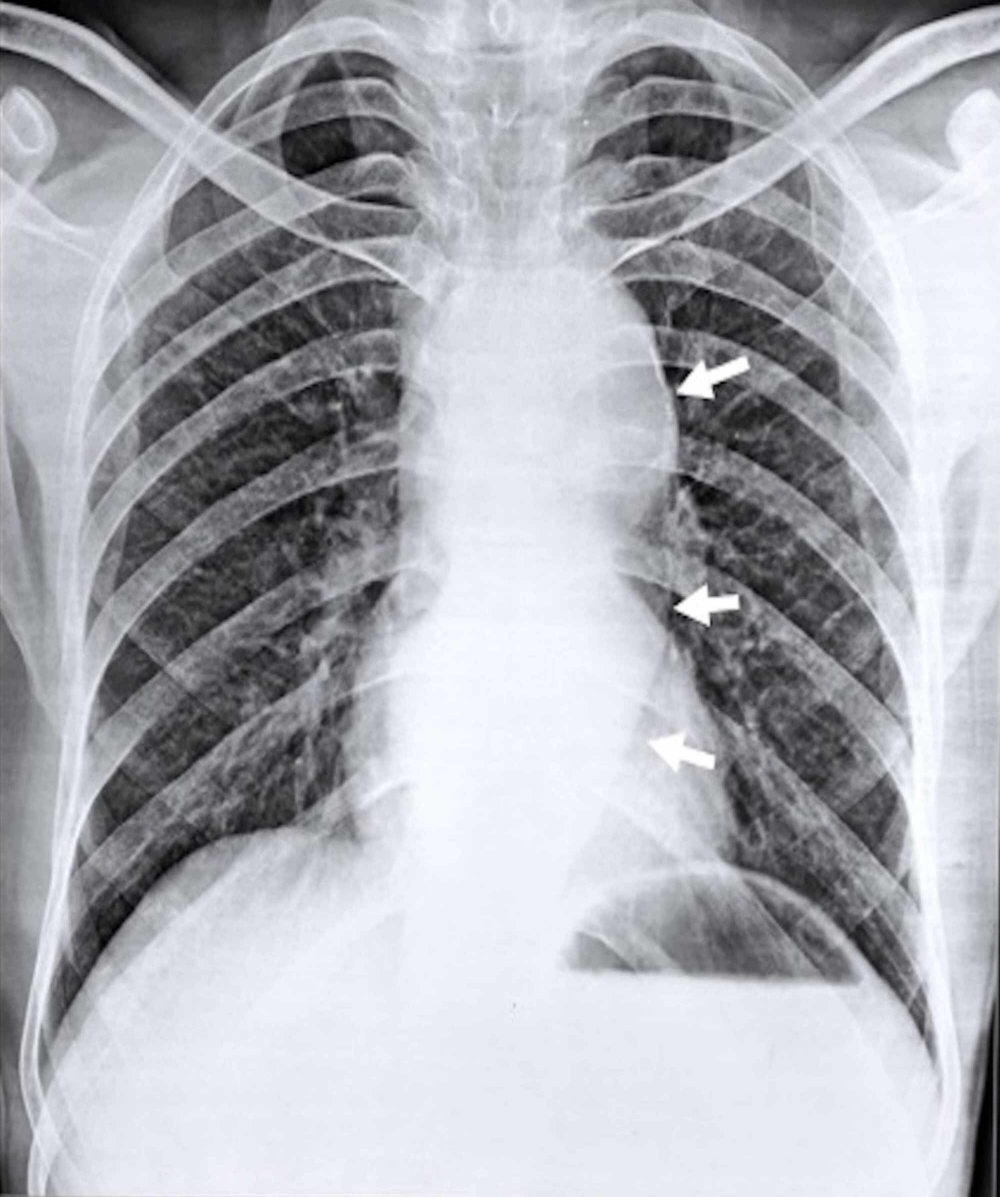
Chest X-ray (CXR) showing radiodense shadow at aortic knuckle and retrocardiac area (white arrows) with evidence of discontinuous linear calcification (upper white arrow).

On physical examination, he was thin built, undernourished with body mass index (BMI) of 16.1 kg/m^2^. Oral cavity was normal. He had a blood pressure of 110/80 mmHg, pulse rate of 80/minute, respiratory rate of 18/minute. All peripheral pulses were present and baseline saturation was 94% at rest. Pallor was present, rest of the examination was normal. A 12-lead-electrocardiogram was normal. Chest X-ray showed radio dense shadows at aortic knuckle and retrocardiac area for which he was referred to cardiology clinic (Figure 2).

**Figure 3:**
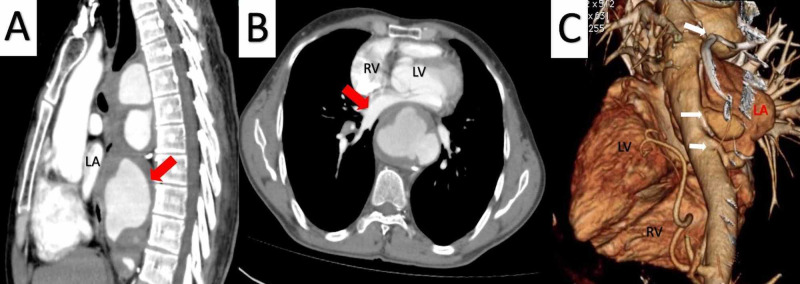
(A) Contrast enhanced sagittal computed tomographic imaging of thorax showing multiple, complex, multilobulated aneurysms arising from arch, continuing to descending thoracic aorta. Lowermost large aneurysm of size 4.9 x 5.2 x 3.4 cm is seen compressing left atrium (LA). Mural thrombosis is noted (Red arrow). (B) Contrast enhanced axial computed tomographic imaging of thorax (D8-D9 vertebra) showing multilobulated wide aneurysm of size 4.9 x 5.2 x 3.4 cm with mural thrombus compressing the left atrium (LA) anteriorly. Red arrow points to left atrium (LA). LV is left ventricle and RV is right ventricle. (C) Volumetric rendered imaging of thorax revealing multiple saccular aneurysms arising from descending aorta (white arrows) in relation to left atrium (LA).

Computed tomography of chest revealed multiple eccentric saccular aneurysm with mural thrombus arising from the arch and descending thoracic aorta, with compression of trachea, oesophagus and left atrium (Figure 3, Video 2).

**Video 2:** Contrast enhanced axial computed tomographic imaging of thorax showing thoracic aneurysm with mural thrombi (white arrow) causing compression of trachea (green arrow), esophagus (blue arrow) and left atrium (red arrow).

**Figure 4:**
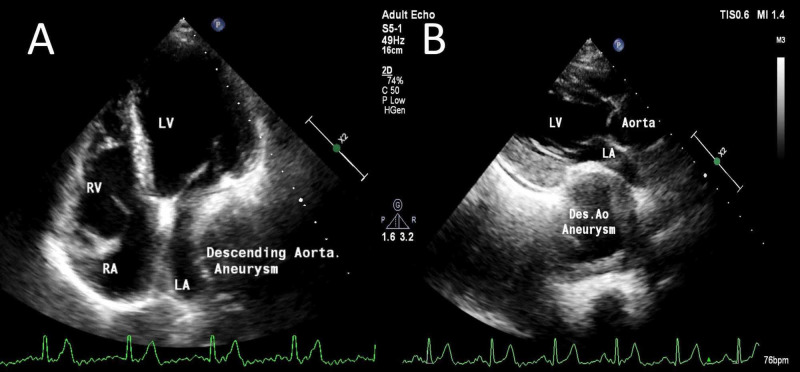
(A) Apical 4 chamber two-dimensional transthoracic echocardiogram showing compression of left atrium by descending thoracic aneurysm. (B) Parasternal long axis view showing compression of left atrium by descending thoracic aneurysm.

Echocardiography showed compression of left atrium by large descending thoracic aneurysm with normal left ventricle systolic function (Figure 4, Video 3).

**Video 3:** 2D echocardiogram (Apical 4 chamber view) showing left atrium (red arrow) compression by large descending thoracic aneurysm (white arrow) with mural thrombus.

Diagnosis of Ortner’s syndrome secondary to compression of left recurrent laryngeal nerve palsy by thoracic aortic aneurysm, as it loops around arch of aorta, was made. Coronary angiography showed normal coronaries. Endovascular aorta aneurysm repair (EVAR) was planned, however in view of financial constraints, it could not take place.

## Discussion

Ortner’s syndrome is a well-known clinical entity. In 1897, Norbert Ortner comprehended that an enlarged auricle caused compression of the LRLN against the aorta resulting in nerve palsy [2]. In 1904, Alexander described an enlarged left pulmonary artery causing compression of the LRLN [3]. In 1911, Fetterolf and Norris demonstrated that a dilated left auricle caused compression of the LRLN between the left pulmonary artery and aorta or ligamentum arteriosum [4].

Currently, cardiovocal syndrome is used to describe LRLN palsy caused by any cardiovascular pathology. Causes include left side heart failure, patent ductus arteriosus, Eisenmenger syndrome, primary pulmonary hypertension, and various other cardiac pathologies [5]. As more cardiovocal syndrome cases were reported, various new explanations of the cause have emerged such as lymphadenitis and scarring in the aortic window causing nerve fixation, pressure from the left bronchus, right ventricular hypertrophy, or pulmonary artery atherosclerosis [6].

Cases of Ortner's syndrome due to aortic aneurysms have been reported in the literature [7-11]. Thoracic aortic aneurysms are usually asymptomatic. When symptomatic, they usually present with chest pain. Hoarseness as a symptom without chest pain in a case of aortic aneurysm is a rare presentation [8]. In this case, the patient presented with hoarseness of voice secondary to compression of the left recurrent laryngeal nerve palsy by saccular aneurysm arising from the thoracic aorta. The nerve palsy is probably due to stretching of the nerve as imaging showed the aneurysm to be in close proximity to the site where the LRLN loops around the aortic arch.

## Conclusions

Ortner’s syndrome, also known as cardiovocal syndrome, is a rare condition, secondary to cardiopulmonary disorders. It is caused by compression of the left recurrent laryngeal nerve between the aorta and pulmonary artery. This report reinforces the importance of performing a cardiovascular workup in cases of unilateral vocal cord palsy when there is no other apparent cause.
